# The performance of ChatGPT and other large language models on multiple‐choice questions in biomedical disciplines: A meta‐analysis

**DOI:** 10.1002/ase.70262

**Published:** 2026-05-19

**Authors:** Colleen M. Cheverko, Volodymyr Mavrych, Olena Bolgova, Fathima Raahima Riyas Mohamed, Jennifer Westrick, Lorena Juarez, Emily Rush, Kathryn A. Solka, Alison F. Doubleday, Jessica N. Byram, Robert Becker, Victoria Gomez, Brenda K. Anak Ganeng, Leslie A. Hoffman, Victoria A. Roach, Kirsten M. Brown, Nicole DeVaul, Colleen N. Garnett, Hannah L. Herriott, Rebecca S. Lufler, Jason C. Mussell, Joy Y. Balta, Michael A. Pascoe, Jason W. Middleton, Sharon Duffy, Georgina C. Stephens, Adam B. Wilson

**Affiliations:** ^1^ Department of Anatomy and Cell Biology Rush University Chicago Illinois USA; ^2^ College of Medicine Alfaisal University Riyadh Kingdom of Saudi Arabia; ^3^ Rush University Medical Center Library Rush University Chicago Illinois USA; ^4^ Academic Affairs Rush University Chicago Illinois USA; ^5^ Department of Microbial Pathogens and Immunity Rush University Chicago Illinois USA; ^6^ Department of Oral Medicine and Diagnostic Sciences University of Illinois Chicago College of Dentistry Chicago Illinois USA; ^7^ Department of Anatomy, Cell Biology & Physiology Indiana University School of Medicine Indianapolis Indiana USA; ^8^ Department of Anatomy, Cell Biology & Physiology Indiana University School of Medicine Fort Wayne Indiana USA; ^9^ Division of Healthcare Simulation Science, Department of Surgery University of Washington Seattle Washington USA; ^10^ Department of Anatomy & Cell Biology The George Washington University School of Medicine and Health Sciences Washington DC USA; ^11^ Department of Cell, Developmental, & Integrative Biology University of Alabama at Birmingham Heersink School of Medicine Birmingham Alabama USA; ^12^ School of Rehabilitative & Health Sciences, Rueckert‐Hartman College of Health Professions Regis University Denver Colorado USA; ^13^ Department of Medical Education Tufts University School of Medicine Boston Massachusetts USA; ^14^ Department of Cell Biology and Anatomy Louisiana State University New Orleans Louisiana USA; ^15^ Anatomy Learning Institute, College of Health Sciences Point Loma Nazarene University San Diego California USA; ^16^ Department of Physical Medicine & Rehabilitation University of Colorado Anschutz Aurora Colorado USA; ^17^ LSU Health New Orleans Libraries Louisiana State University New Orleans Louisiana USA; ^18^ Department of Anatomy and Developmental Biology Monash University Clayton Victoria Australia

**Keywords:** accuracy, artificial intelligence, large language models, multiple‐choice questions

## Abstract

While large language models (LLMs) have shown promise as learning tools for medical education, their reported accuracy on multiple‐choice questions (MCQs) varies widely across studies, necessitating synthesis. This meta‐analysis synthesizes LLM accuracy on text‐based MCQs from biomedical disciplines and USMLE Step 1‐level content and explores study characteristics that moderate LLM performance. Studies published between January 1, 2022, and August 5, 2025, were identified using seven databases. Titles and abstracts were screened against eligibility criteria, which included testing LLM performance on text‐based MCQs in English. Extracted data included accuracy rates (correct responses/total questions) and study characteristics. A random‐effects proportional meta‐analysis was used to calculate the pooled accuracy of LLM versions across studies, and a meta‐regression tested the effects of study characteristics on accuracy. The final analysis included 41 articles comprising 207 proportions. Newer OpenAI models (GPT‐4o through GPT o1 = 90%; GPT‐4 = 82%) demonstrated significantly higher accuracy than earlier versions (GPT‐3/3.5 = 59%; *p* < 0.001). Model version explained 56% of the total variance (*p* < 0.001). The pooled accuracy of newer Anthropic (Claude 3 through 3.7 = 86%) and DeepSeek (R1 = 86%) versions fell within the range of performance for the newer OpenAI versions, while newer Google (Gemini series = 76%) and Microsoft (Copilot = 69%) versions demonstrated lower performance. A discipline‐level analysis demonstrated that OpenAI's GPT‐4 through GPT‐o1 models achieved pooled accuracy rates between 83 and 87% for most disciplines, including the anatomical sciences. These findings demonstrate that newer LLMs are achieving higher accuracy than older models on biomedical and Step 1‐level MCQs, supporting their potential integration as supplementary study tools in foundational biomedical education.

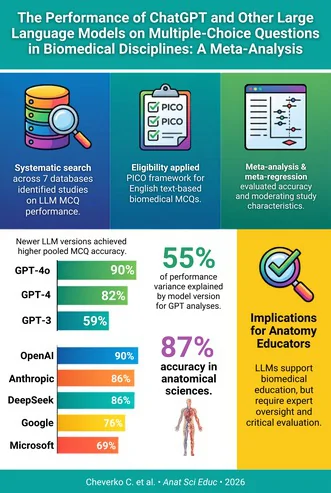

## INTRODUCTION

Large language models (LLMs) are fundamentally transforming medical education. They are driving a paradigm shift toward a model of learning and teaching that provides instant feedback, realistic clinical simulations, and tailored educational resources.^1^, ^2^, ^3^, ^4^, ^5^, ^6^ Learners and educators are increasingly leveraging LLMs to generate and answer multiple‐choice questions (MCQs) as study aids and for formative assessment.^7^, ^8^, ^9^ However, before LLMs can be confidently integrated into educational settings, a comprehensive synthesis of evidence comparing LLM companies and versions is needed to understand their accuracy.

While widespread interest in LLM applications for pre‐clerkship medical education has spurred numerous studies testing their performance on MCQ datasets, these studies reveal substantial variation in accuracy. For example, in pathology, GPT‐3.5's accuracy ranges from 40% to 75%,^10^, ^11^ while GPT‐4's spans from 57% to 81%.^12^, ^13^ LLM performance in gross anatomy (GPT‐3.5: 42%–80%^14^, ^15^; GPT‐4: 62%–100%^14^, ^16^) and physiology (GPT‐3.5: 47%–80%^15^, ^17^; GPT‐4: 50%–92%^10^, ^18^) has demonstrated a similar wide range of accuracy. Wide within‐discipline accuracy ranges suggest that factors beyond subject matter (e.g., question complexity, question source, inclusion of imaging questions, and web accessibility) may influence LLM performance. However, no meta‐analysis to date has quantified these effects or examined temporal trends in LLM accuracy on biomedical MCQ datasets.

Prior meta‐analyses have focused on medical specialties such as ophthalmology and surgery,^19^, ^20^, ^21^ whose findings may not be directly transferable to foundational biomedical education. Related meta‐analyses of medical licensing examinations worldwide have yielded variable results that are similar to those of discipline‐specific studies, with pooled accuracies ranging from 58% to 82%, depending on the LLM version and the included examinations.^22^, ^23^, ^24^, ^25^ The heterogeneity of these outcomes may be a consequence of analyzing the USMLE Step 1, 2CK, and 3 datasets collectively, despite their intended use to assess different academic levels. Moreover, these and other meta‐analyses have been limited by focusing exclusively on ChatGPT's performance,^22^, ^23^, ^24^, ^25^ and few have leveraged meta‐regressions to quantify performance improvements in LLM versions over time.

This systematic review and meta‐analysis addresses these gaps by summarizing LLM performance on text‐based MCQs in biomedical disciplines assessed on the USMLE Step 1 examination. This study seeks to answer the following research questions: (1) How have LLM version updates influenced their performance accuracy in answering biomedical‐focused MCQs; (2) How do different moderators (e.g., question type, stem type, MCQ source, and probability of web access) affect the pooled accuracy of LLMs; and (3) Is LLM accuracy consistent across USMLE biomedical disciplines? This multidisciplinary synthesis of temporal trends and moderators enables educators in the anatomical sciences to benchmark LLM performance against other USMLE Step 1 disciplines, providing a comparative framework that is essential for evidence‐based AI integration within increasingly integrated medical curricula.

## METHODS

This systematic review and meta‐analysis followed the Preferred Reporting Items for Systematic Reviews and Meta‐Analyses (PRISMA) guidelines.^26^ Institutional Review Board approval was not required since no human subjects participated directly in this research.

### Study design

This biomedical‐focused analysis represents one aspect of a multidisciplinary investigation examining LLM performance across health care fields. We conducted a unified literature search across medical (biomedical and clinical), dental, and allied health fields to identify disciplines with sufficient study volume for meta‐analysis. All identified records were processed through a standardized workflow encompassing title and abstract screening, full‐text review, and data extraction, irrespective of disciplinary origin. Findings specific to non‐biomedical specialties (e.g., clerkship and clinical‐focused studies) are reported in a separate manuscript.

We developed a comprehensive search strategy in consultation with medical librarians (JW and LJ). We systematically searched PubMed/MEDLINE, Scopus, Embase, CINAHL, PsycInfo, ERIC, and Google Scholar databases for records published between January 1, 2022 and our search date of August 5, 2025. The search protocol integrated controlled vocabularies (including MeSH terms) and keywords such as MCQ assessments and educational examinations. We also included additional studies located through reference checking. Reproducible search protocols are provided in Appendix S1.

### Eligibility criteria

Study selection utilized the PICO framework^27^ (Population, Intervention, Comparison, Outcomes). Our population [P] comprised single‐best‐answer MCQs used in biomedical disciplines or USMLE Step 1‐style assessments. Studies reporting LLM performance in clinical specialties, veterinary medicine, allied health disciplines, or dentistry were excluded. The intervention [I] required empirical testing of LLM performance on biomedical MCQs. While many studies compared [C] multiple LLM versions, such comparisons were not mandatory for inclusion. Accuracy outcomes [O] were operationalized as the number of correct responses divided by the total number of MCQs administered.

Studies qualified for inclusion if they (1) published empirical research in English, (2) satisfied the PICO criteria described above, and (3) provided adequate methodological detail for the extraction of contextual and numerical data. No geographic limitations were imposed; however, the MCQs had to be administered in English. Results from translations of MCQs into English were included if the authors reported human verification of the translations before entering the questions into the LLM(s). Studies, or subsets of studies, that administered non‐English questions were excluded due to potential language‐based performance differences. Additional exclusion criteria included non‐refereed preprints, retracted articles, inaccessible articles, and publication types such as literature reviews, conference abstracts, and theses and dissertations. Corresponding authors were contacted as needed to acquire missing information or to clarify ambiguous reporting.

### Record screening and inter‐rater agreement

Covidence systematic review software (Covidence, Veritas Health Innovation, Melbourne, Australia; www.covidence.org) was used to manage title and abstract screening, full‐text reviews, and data extraction. Two investigators independently reviewed all titles and abstracts, with screening responsibilities distributed among five team members (ABW, CMC, VM, OB, ER). Records were advanced to full‐text review when both reviewers agreed on inclusion. Screening disagreements were reviewed by a third reviewer, who determined whether the record should proceed to full‐text review. Inter‐rater reliability was measured using Cohen's *κ* statistic to evaluate the consistency of screening decisions. Cohen's *κ* interpretation followed established benchmarks (0.21–0.40 = fair, 0.41–0.60 = moderate, 0.61–0.80 = substantial, and 0.81–1.00 = almost perfect agreement).^28^

### Full‐text review and data extraction

Full‐text evaluation and data extraction were conducted simultaneously. In this step, key variables were extracted, including general study characteristics and relevant methodological details (Table 1). After frame‐of‐reference training, 23 investigators shared responsibilities for data extraction. Two reviewers independently extracted data from each record using a standardized form developed within Covidence. A third reviewer resolved data extraction disagreements.

**TABLE 1:** Summary of variables included for analysis.

Variable	Definitions, selection options, or examples	Inclusion criteria
LLM company name and version	E.g., OpenAI: GPT‐4; Anthropic: Claude 3.5 Sonnet; Microsoft: Copilot; Google: Gemini Flash 2.0	Analysis included any LLM company tested in 5+ studies
Numerator	The number of questions answered correctly	N/A
Denominator	The number of questions input into the LLM	Data points were included if ≥5 MCQs were input into the LLM
Biomedical discipline	Indicates the most fitting categorization of each question set. “USMLE Step 1/Equivalent” was used to note studies that aggregated USMLE Step 1 questions and included foreign exam equivalencies.	Disciplines with 5+ data points were included in this study.
Training priming and fine‐tuning	Indication of whether the LLM was trained, primed, or fine‐tuned before answering the MCQs	Only studies that used commercial LLMs without training, priming, or fine‐tuning were included for analysis. Pretraining runs were included in the analysis when data could be extracted separately from post‐training runs
Timepoint	The number of times the tested MCQs were run through a single LLM. Options: Single/first timepoint Multiple timepoints with data for each Average of multiple timepoints	Only single/first timepoint data were included in analysis to eliminate temporal confounds, avoid aggregation bias, ensure methodological uniformity across studies with various research designs, and most closely represent student use
Prompt type	Indicates whether prompting quality was tested alongside MCQ analysis	Only studies that used or were inferred to use zero‐shot prompting were included in analyses. Pre‐prompting runs were included in the analysis when data could be extracted from post‐training runs
Question type^a^	Indicates the type of input for the MCQs. Options: MCQs only MCQs + other (e.g., image‐based MCQs)	Data for text‐based, single‐best answer MCQs without images were prioritized and included. MCQ + other was included only when outcomes for mixed question types could not be separated
Stem type^a^	Indicates the type of MCQ inputted by researchers for each study Options: Question/stem only Vignette + question/stem Mixed Not specified	When provided, question stems were evaluated in tables, figures, or supplemental materials
MCQ source^a^	Indicates the source or origin of the MCQs used in each study Options: Affiliated with an association or authoritative body Locally developed For‐profit commercial product (e.g., textbooks) Free resource (e.g., online question bank) Not specified	Author statements about question source were evaluated to discern these categories
Inferred likelihood of web accessibility^a^	Indicates the likelihood that an LLM version with web‐scanning capabilities could find the questions set Options: Unknown Low High	Web accessibility was inferred based on contextual clues if not directly reported in the study (e.g., locally developed MCQs were inferred to have a lower probability of web accessibility than a free online question bank)

^a^ Indicates that a variable was explored as a covariate using meta‐regression.

### Quality appraisal

Commonly used instruments to explore methodological quality, such as the Medical Education Research Study Quality Instrument (MERSQI), did not apply to the design or objectives of this study. Therefore, the methodological quality of included studies was not assessed.

### Statistical analyses

Data were organized using Microsoft Excel (version 2306, Microsoft Corporation, Redmond, WA). Descriptive statistics werereported to characterize study features. Meta‐analyses were conducted using R statistical software (version 4.3.2, “meta” package^29^). A random‐effects proportional meta‐analysis employing a logit transformation and inverse‐variance weighting was used to analyze the number of correct answers relative to the total number of MCQs across studies and LLM versions.^30^, ^31^ Results are presented as pooled proportions with two‐sided confidence intervals (CIs) at 95%. Between‐study heterogeneity was assessed using the *Q*‐statistic,^32^ and the proportion of variance attributable to study‐level differences was assessed using the *I* ^2^ statistic.^33^ *I* ^2^ values <25% were interpreted as negligible heterogeneity, while *I* ^2^ values >75% were interpreted as substantial heterogeneity.^34^ Publication bias testing was omitted because there is insufficient evidence to justify its use in proportional meta‐analyses.^35^

Meta‐regression was used to examine potential sources of heterogeneity and to assess version effects across OpenAI's LLMs, using the pooled, all‐discipline data.^36^ All meta‐regressions employed maximum‐likelihood estimation with a random‐effects model. OpenAI LLM versions were explored as covariates using the following groupings: GPT‐3/3.5, GPT‐4, and GPT‐4o through GPT o1 (henceforth GPT‐4o_o1). Question type, stem type, and MCQ source (see Table 1) were evaluated as potential covariates for explaining the observed variance in LLM accuracy across studies. The likelihood of MCQ answers being retrievable from the internet was included as a potential covariate for newer LLMs capable of web browsing (e.g., OpenAI's GPT‐4 or newer).

Discipline‐level analyses included disciplines testing OpenAI's LLMs in ≥5 studies. Included proportions were divided into two groups: (1) GPT‐3/3.5, representing earlier LLM versions, and (2) GPT‐4 through GPT o1 (henceforth GPT‐4_o1), representing more recent LLM versions. Relatively small sample sizes for GPT‐4o through GPT o1 versions precluded separating this category from GPT‐4 for discipline‐specific analyses. Meta‐regressions were not conducted because of small sample sizes.

## RESULTS

### Search and screening outcomes

**FIGURE 1:**
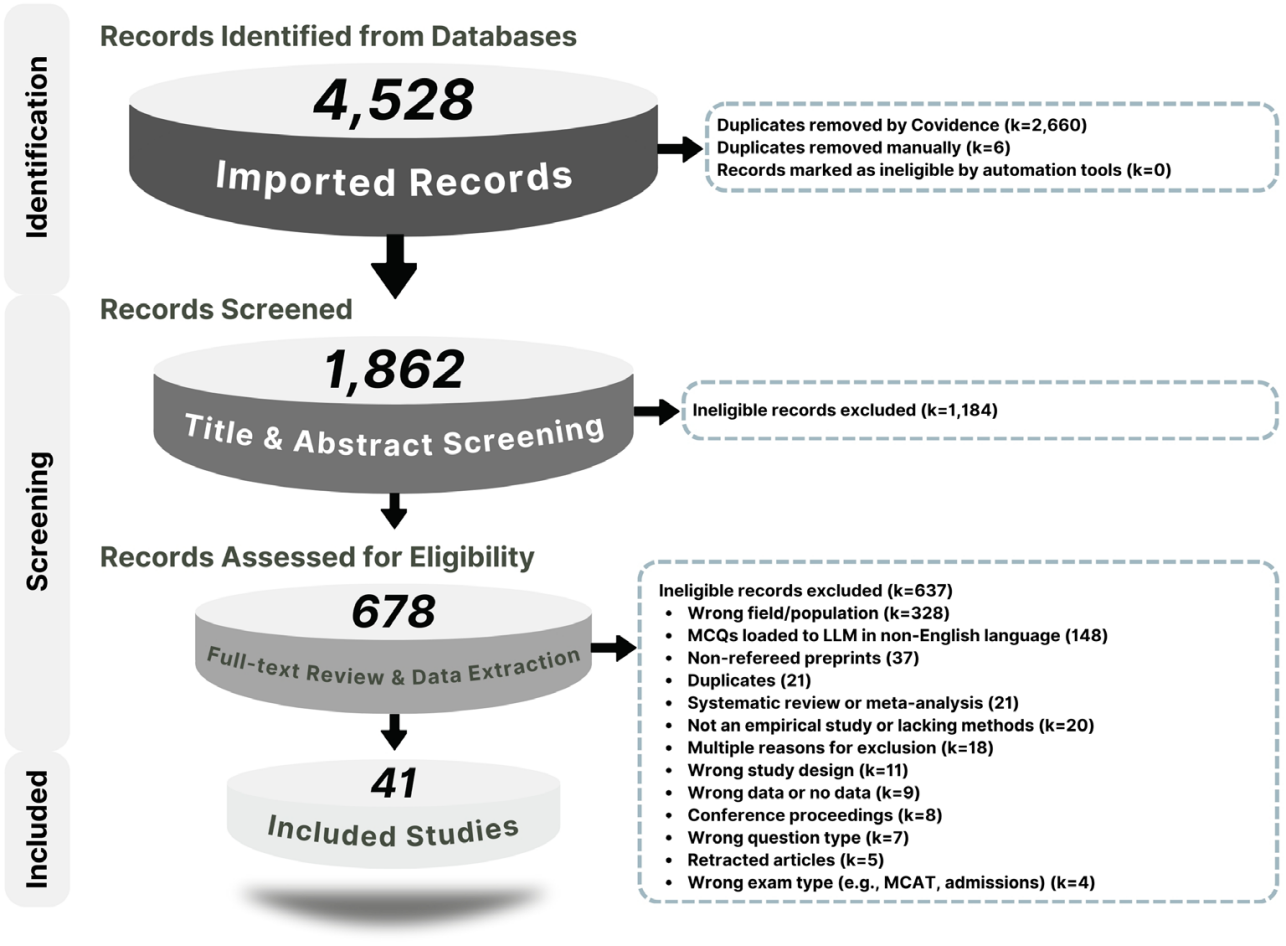
Diagram of the study inclusion process.

After removing duplicates, the literature search across medical, dental, and allied health disciplines identified 1862 unique records. Of these, 678 studies were advanced to full‐text evaluation, and 41 studies met the inclusion criteria (Figure 1). Inter‐rater reliability for title and abstract screening demonstrated substantial agreement (mean *κ* = 0.78, range = 0.63–0.93) across the five independent investigators.

### Summary of study characteristics

The 41 studies included 207 proportions. These studies explored LLM performance in the disciplines of anatomical sciences (i.e., gross anatomy, embryology, histology, cell biology, and neuroscience; 31.7%, with data present in 13 of 41 studies), biochemistry (22.0%, 9/41), microbiology and immunology (12.2%, 5/41), pathology (22.0%, 9/41), pharmacology (14.6%, 6/41), and physiology (19.5%, 8/41). Several studies (19.5%, 8/41) reported outcomes for multiple disciplines. Fifteen studies (36.6%) investigated MCQs focused on USMLE Step 1 or equivalent examinations without subdividing results by discipline (henceforth “USMLE Step 1/Equivalent”). Disciplines represented by <5 studies [i.e., behavioral sciences (2/41), bioethics (2/41), biostatistics or statistics (1/41), and genetics (1/41)] were included in analyses of the full dataset but excluded from discipline‐level analyses because of insufficient sample sizes.

Most corresponding authors were affiliated with institutions in countries where English is considered a primary language (53.1%, 23/41). Nearly all studies (97.6%, 40/41) tested the accuracy of OpenAI's LLMs, and 29.3% (12/41) reported results from other AI companies. MCQs were sourced from locally developed questions, resources affiliated with associations or authoritative bodies, for‐profit commercial products (e.g., textbooks), and/or free resources (e.g., web‐based question banks). The number of MCQs tested across studies ranged from 5 to 2377 (mean = 87; median = 31). Appendix S2 reports detailed study characteristics.

### Meta‐analytic outcomes

OpenAI's LLM accuracy on biomedical MCQs markedly improved across its successive versions. Meta‐analytic findings revealed that GPT‐4o_o1 versions achieved a 90.1% pooled accuracy (CI: 88.0%–91.9%), representing a statistically significant advancement (*p* < 0.001) from previous versions [GPT‐4 = 81.9% (CI: 78.4%–85.0%); GPT‐3/3.5 = 59.3% (CI: 54.2%–64.3%)]. Forest plots detailing OpenAI LLM version results are included in Appendix S3 (Figures S3.1–S3.3). A meta‐regression analysis of OpenAI's GPT versions indicated that version progression explained 54.9% of the observed variance in performance (*p* < 0.001). When benchmarked against GPT‐3/3.5, GPT‐4 exhibited an elevated tendency for correct responses (*β* = 1.14, *p* < 0.001), yielding an odds ratio of 3.13 (i.e., GPT‐4 was approximately three times more likely to answer correctly than GPT‐3/3.5). GPT‐4o_o1 versions demonstrated larger gains (*β* = 1.85, *p* < 0.001), yielding an odds ratio of 6.36, indicating that they were more than six times as likely to produce accurate MCQ responses than GPT‐3/3.5. These findings demonstrate a clear trajectory of enhanced capabilities across model iterations.

Additional meta‐regressions were conducted on the entire OpenAI dataset (including all disciplines and all studies using OpenAI LLMs) to determine whether covariates (question type, stem type, MCQ source, and inferred likelihood of web access) explained portions of the observed heterogeneity. To ensure analytical precision, analyses were restricted to studies with clearly defined or quantifiable data within each category (i.e., studies with unknown or mixed classifications were excluded for that variable in the meta‐regression). Under these conditions, only the LLM versions described above explained portions of the observed heterogeneity. None of the additional covariates independently explained more than 3% of the heterogeneity observed in performance accuracy (*p* = 0.09–0.95).

### Discipline‐specific performance

**FIGURE 2:**
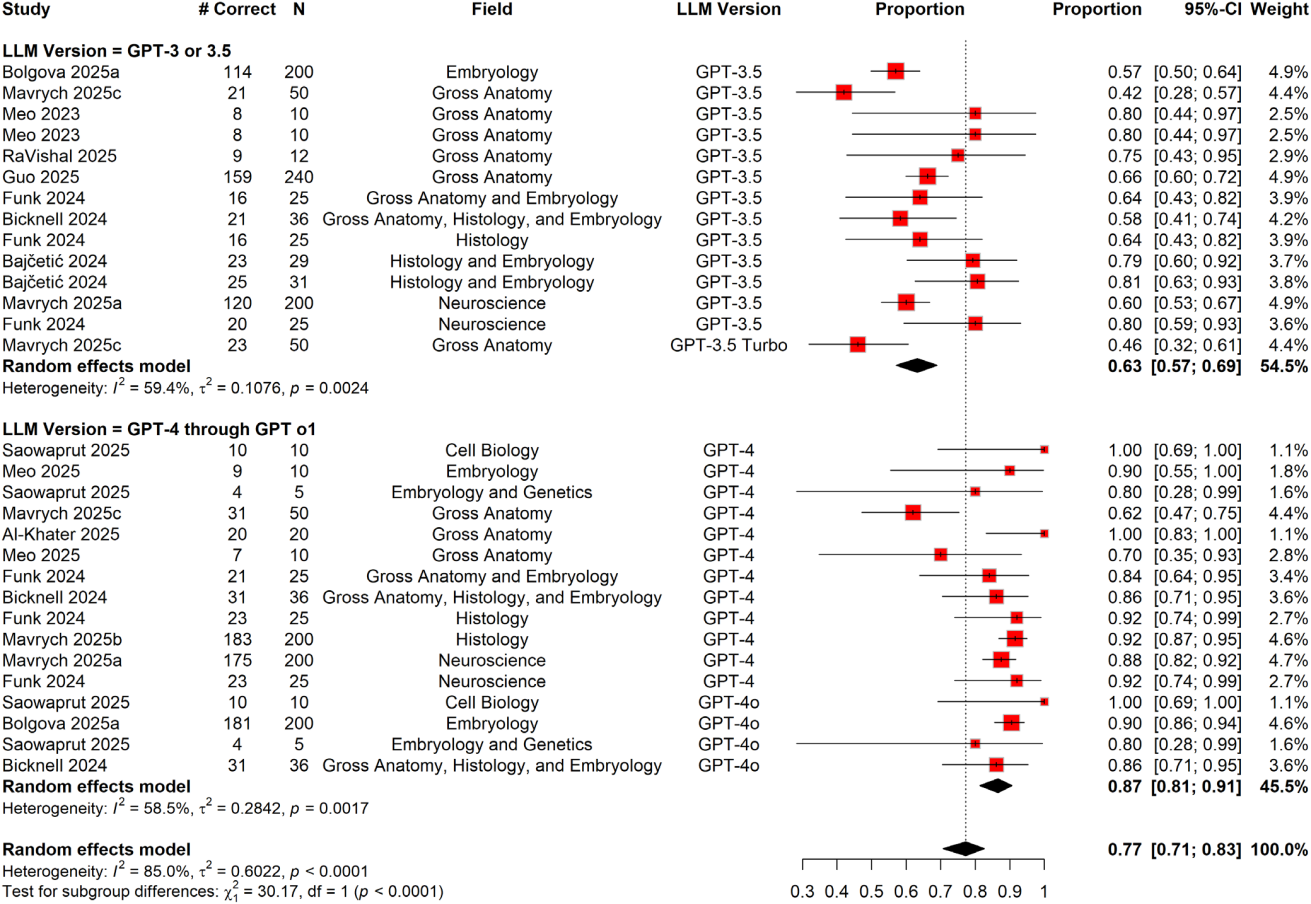
OpenAI results for the anatomical sciences. Subdisciplines are listed for reference. These subdisciplines were defined by the authors of each included study, and datasets could not be further subdivided.

**FIGURE 3:**
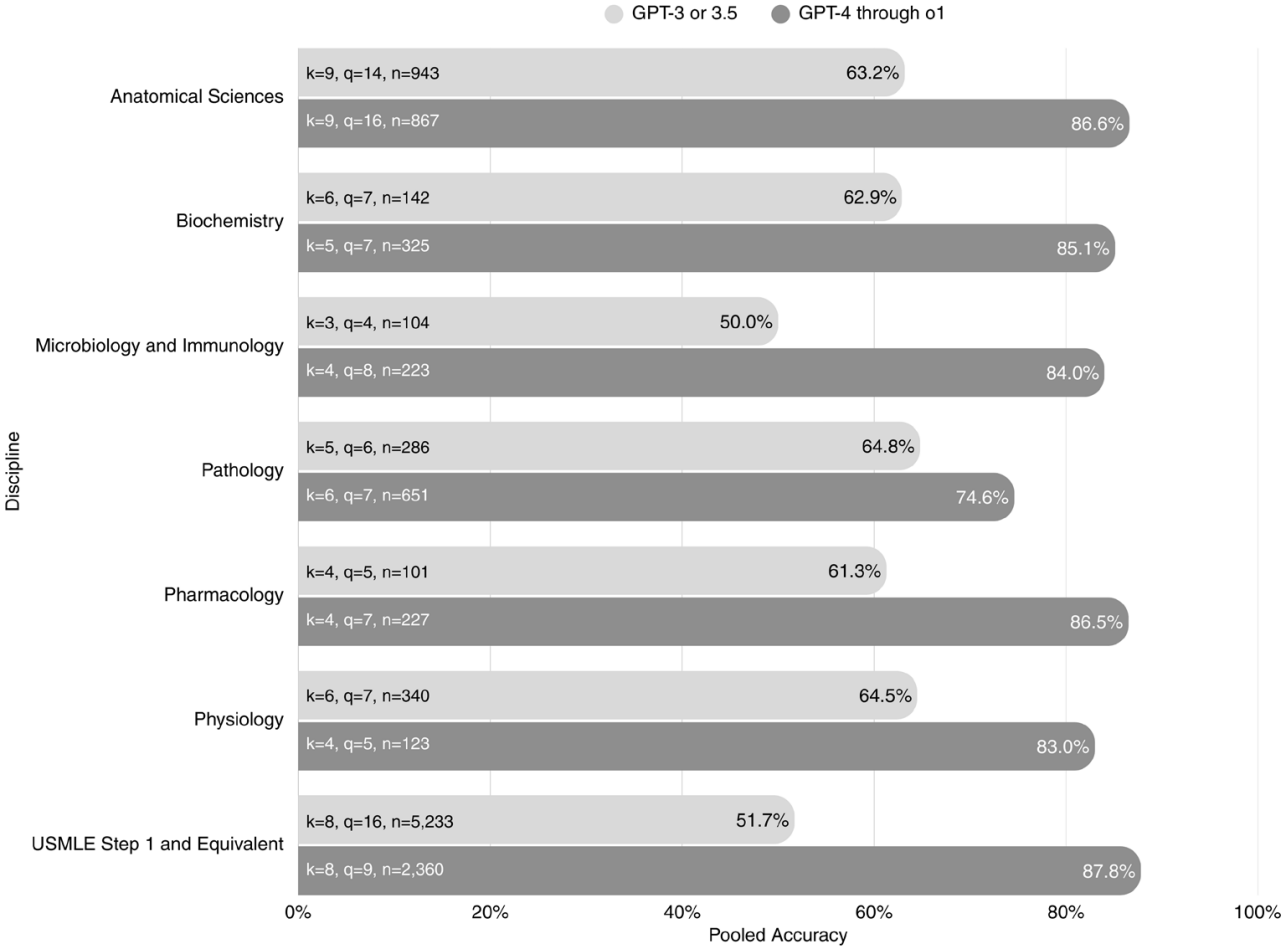
Pooled accuracy on MCQs by discipline and OpenAI LLM version. *K* represents the number of studies investigating each discipline, and *Q* represents the number of question sets investigated. *N* represents the total number of MCQs investigated. Forest plots with 95% confidence intervals for disciplines and LLM versions are presented in Supplemental Appendix S3 (Figures S3.4–S3.9), and a detailed breakdown of results is presented in Supplemental Appendix S4 (Table S4.1).

GPT‐4_o1 versions achieved a pooled accuracy of 87% for the anatomical sciences, compared with 83% for GPT‐3/3.5 (Figure 2). GPT‐4_o1 demonstrated a pooled accuracy of ≥83% for “USMLE Step 1/Equivalent” and four other biomedical disciplines (Figure 3). GPT‐4_o1 versions achieved 75% accuracy in pathology. Using meta‐regression, this difference between pathology and the other disciplines was statistically significant (*p* = 0.04). Across all disciplines, the pooled accuracy of GPT‐4_o1 versions was higher than that of older versions (GPT‐3/3.5: pooled accuracy ≤65.0%; Figure 3). Forest plots depicting pooled accuracy and CIs for each discipline are provided in Supplemental Appendix S3 (Figures S3.4–S3.9). Additional sources of heterogeneity could not be explored by discipline because of small sample sizes.

### GenAI company performance

**FIGURE 4:**
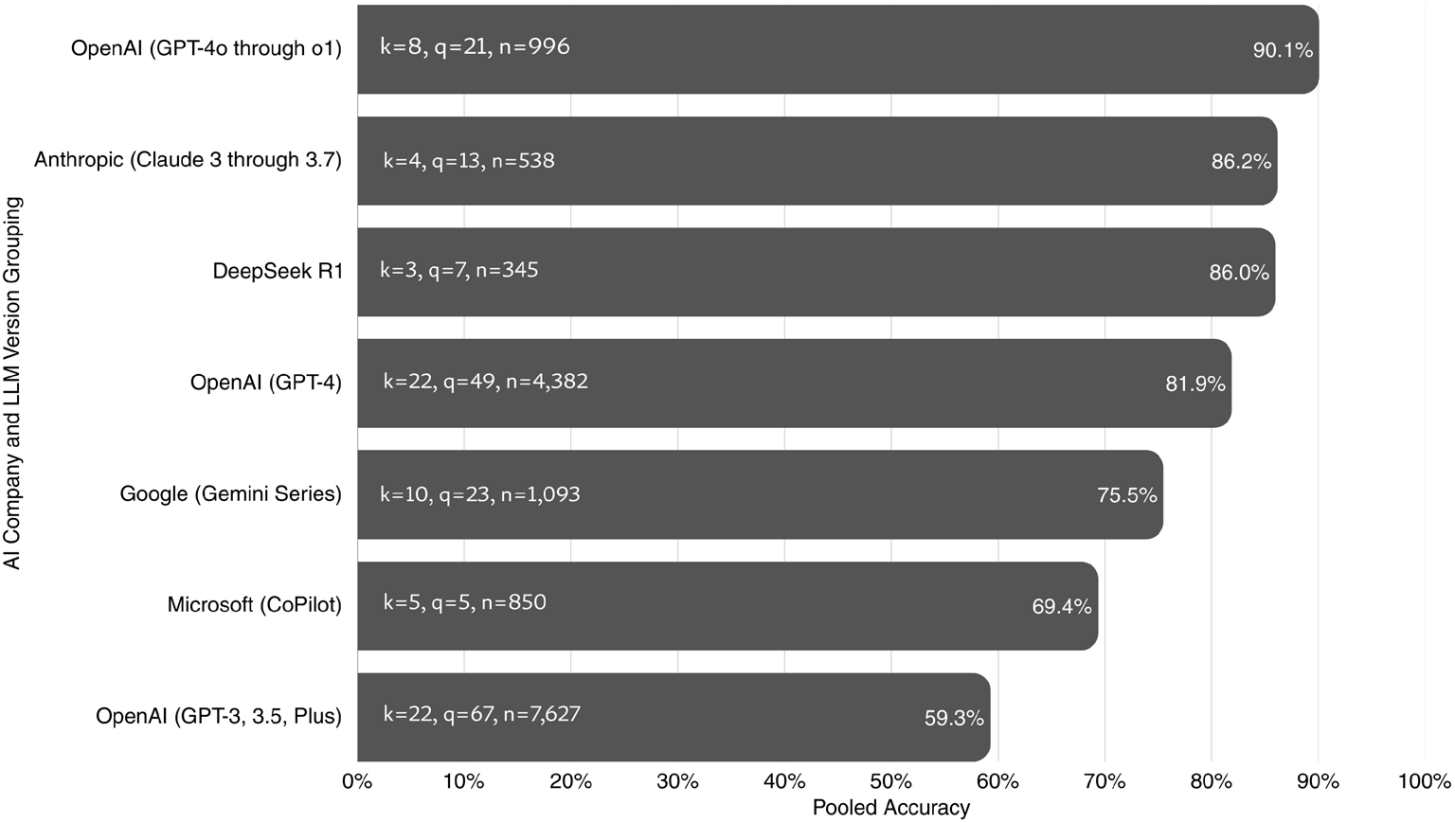
Pooled accuracy of MCQs by newer versions of Anthropic, DeepSeek, Google, and Microsoft. OpenAI values are provided for comparison. *K* represents the number of studies including data for each LLM version, *Q* represents the number of question sets investigated, and *N* represents the total number of MCQs investigated. A forest plot with 95% confidence intervals for LLM versions is presented in Supplemental Appendix S3 (Figure S3.10), and a detailed breakdown of the results is presented in Supplemental Appendix S4 (Table S4.2). Please note that the smaller number of studies investigating DeepSeek, Anthropic, Google, and Microsoft performance compared with that of OpenAI increases the uncertainty of the confidence intervals for these models.

The performance of newer LLM models from other companies with ≥5 data points was compared with that of OpenAI's models. Anthropic's Claude 3 through 3.7 (86.2%, CI = 81.7%–89.7%) and DeepSeek's R1 (86.0%, CI = 78.0%–90.3%) models modestly underperformed compared with OpenAI's GPT‐4o_o1 models. The performance of the Google Gemini series (75.5%, CI = 68.0%–81.7%) and Microsoft Copilot (69.4%, CI = 54.0%–81.4%) trailed that of the leading companies (Figure 4). Heterogeneity was high across Google Gemini and Microsoft Copilot (*I* ^2^ ≥ 76%) but lower for Anthropic and DeepSeek (*I* ^2^ ≤ 20%). Insufficient sample sizes precluded sub‐analyses or meta‐regressions to further explore differences in heterogeneity. Likewise, discipline‐specific performance could not be explored for these models due to small sample sizes.

## DISCUSSION

This meta‐analysis evaluated the progression of OpenAI's LLMs in answering biomedical MCQs and benchmarked their performance against other leading LLMs. Accuracy significantly improved with each successive OpenAI model. Newer GPT versions (GPT 4o through GPT o1; henceforth “GPT4o_o1”; 90%) surpassed both GPT‐4 (82%) and GPT‐3/3.5 (59%). LLM version progression accounted for 54.9% of the observed performance variance, with GPT‐4o_o1 versions exhibiting substantially higher odds of correct responses than earlier models. These outcomes suggest a trajectory of enhanced capability with each new OpenAI LLM iteration and highlight that LLM version was the primary driver of performance gains in biomedical MCQ tasks.

### Comparisons with the existing literature

Existing meta‐analyses exploring LLM accuracy on MCQs in healthcare education are predominantly focused on content from medical specialties, dentistry, or licensing examinations. For example, the pooled accuracy of GPT‐3.5 across four meta‐analyses in medical specialties or dentistry ranged from 52% to 62%.^19^, ^20^, ^21^ GPT‐4's accuracy among these same studies ranged from 73% to 77%.^19^, ^20^, ^21^ Our accuracy outcomes for biomedical MCQs answered by GPT‐3/3.5 (59%) fell within this range. However, our GPT‐4 outcomes (82%) were modestly higher than those previously reported for medicine or dentistry, possibly because of differences in topical foci or our larger sample size of included studies (*k* = 41 compared with *k* ≤ 29 in prior medical specialty or dental meta‐analyses).^19^, ^20^, ^21^

Meta‐analyses summarizing LLM accuracy on health professions licensing examinations have reported pooled accuracies of 58%–61% for GPT‐3.5 and 80%–82% for GPT‐4.^22^, ^23^, ^24^, ^25^ Our GPT‐3.5 and GPT‐4 outcomes fell within these narrow ranges, suggesting congruence between LLM capabilities on licensing examinations and biomedical MCQs. At the time of this study, no other meta‐analyses had examined the accuracy of OpenAI's GPT‐4o or newer versions on medical licensing exams.

Literature reviews comparing AI companies' performance have consistently shown that OpenAI outperforms its competitors.^19^, ^20^, ^37^ Our results align with the existing literature, demonstrating that OpenAI's newer models were more accurate than competing LLMs (Figure 4), though with smaller differences relative to Anthropic and DeepSeek. OpenAI's LLMs have also demonstrated marked improvements over time. Per our findings, GPT‐4o_o1 versions were more than six times as likely to produce accurate MCQ responses (*β* = 1.85, *p* < 0.001, OR = 6.36) than GPT‐3/3.5. In a similar meta‐analysis of radiology subspecialties, GPT‐4o was reported to be 1.65 times more accurate than GPT‐3.5 (*β* = 0.502, *p* < 0.001, OR = 1.65).^38^ This lower magnitude of improvement relative to our findings is expected, given that Nguyen et al.'s inputs contained a higher proportion of image‐based MCQs (47%; 7/15 studies), which were negatively associated with accuracy compared with text‐based MCQs.^38^

### Anatomical sciences performance in context

The pooled accuracy of anatomical sciences MCQs was 83% for GPT‐3/3.5 and 87% for GPT‐4 through GPT o1 (GPT‐4_o1), demonstrating consistent improvements across the aggregated data. These results reflect text‐based MCQs. Most studies that investigated LLM performance on anatomical sciences MCQs excluded images,^10^, ^14^, ^15^, ^18^, ^39^, ^40^, ^41^, ^42^, ^43^, ^44^ but the few studies that included images consistently reported lower accuracy on image‐based MCQs compared with text‐based questions.^16^, ^45^ Likewise, recent studies investigating other aspects of AI performance in image interpretation across anatomy and related disciplines consistently report lower AI performance.^46^, ^47^, ^48^ While image‐based questions were outside the scope of this meta‐analysis, future research could further quantify differences in LLM performance on text‐ versus image‐based MCQs.

Unfortunately, subdiscipline‐specific results could not be further explored because the extracted manuscripts inconsistently combined results from different subdisciplines. For example, some studies reported outcomes for each subdiscipline (e.g., gross anatomy vs. histology), whereas others reported a single outcome across combinations of two or more subdisciplines (e.g., gross anatomy and histology combined). Future studies in the anatomical sciences would benefit from individual reporting of results to enable more granular comparisons across subdisciplines, thereby helping educators apply results to their specific contexts.

Analyses of remaining USMLE Step 1 disciplines revealed consistent LLM performance across most biomedical subjects, including the anatomical sciences, with four other disciplines achieving 83%–87% pooled accuracy for GPT‐4_o1 models. The lower LLM performance in pathology (GPT‐4 = 75%) likely cannot be explained by study moderators because of small sample sizes and high variability in study designs within and between disciplines. We speculate that the lower performance of LLMs in pathology could be influenced by LLM training data limitations. Pathology's reliance on image interpretation and visual pattern recognition may limit access to text‐based training resources. Additionally, pathology questions often integrate information from other disciplines, adding greater complexity than questions testing information from a single discipline.

### Practical implications

While OpenAI's LLMs improved substantially in accuracy across successive releases, educators likely differ in their opinions about what the acceptable threshold should be for classroom implementation, with some more willing than others to adopt AI tools at current accuracy levels for certain activities. In our sample, newer OpenAI models (GPT‐4o through GPT o1) outperformed contemporaneous offerings from Microsoft, Google, Anthropic, and DeepSeek, though Anthropic and DeepSeek demonstrated comparable performance. Our results complement findings from other studies and suggest that scholarly discourse should extend beyond explorations of LLM performance metrics to include evidence‐based implementation strategies in medical education.^49^ While LLM use can be integrated into problem‐based, case‐based, and team‐based learning,^50^, ^51^ MCQ‐based applications are of particular interest for facilitating personalized learning (e.g., providing immediate feedback and generating practice questions) and creating assessments (e.g., drafting case vignettes).^50^ Within these areas, the present findings may be most applicable to low‐stakes formative uses, such as self‐testing, feedback, and faculty‐reviewed practice question generation instead of higher stakes assessment contexts. Given the performance variability observed between disciplines in our meta‐analysis, educators should consult discipline‐specific benchmarking data when determining appropriate educational applications and recommendations.

In the current AI landscape, it is recommended that educators frame LLM use as a tool to support the development of lifelong critical thinking skills rather than as a source of alternative, authoritative answers. With this aim, educators may need to model discipline‐appropriate best practices for LLM use, including how to frame prompts, interpret uncertainty, and verify outputs against authoritative sources. When prompting an LLM to generate or answer MCQs, learners should be encouraged to request answer rationales so they can evaluate the quality of the LLM's reasoning, identify potential errors or oversimplifications, and compare LLM responses with authoritative sources (e.g., medical textbooks). Educators are also encouraged to devise mechanisms or processes that allow students to verify the accuracy of LLMs by consulting content experts. This approach transforms passive answer‐checking into active knowledge construction. Consistent with prior recommendations,^50^, ^52^, ^53^ this approach also centers expert guidance and authoritative resources within the process.

Finally, administrators making LLM subscription decisions should consider MCQ performance alongside other instructionally relevant tasks to identify a platform that best aligns with institutional needs. As AI capabilities evolve, administrators and educators must provide ongoing guidance on appropriate use cases, known limitations, and ethical considerations, while continuously refining policies governing AI use in educational contexts.^54^ Developing institution‐ and domain‐specific benchmarks that reflect local curricular priorities and stakeholder needs may help administrators make more informed platform decisions and guide the iterative review of AI tools over time.

### Limitations

This study was subject to limitations inherent to meta‐analyses, including the potential for selection biases and incomplete reporting.^31^ To minimize translation‐related accuracy confounds, we included only studies that submitted English‐language questions, which may have led to the omission of relevant data and limited the generalizability of our findings to English‐speaking contexts. Although we included only studies with zero‐shot prompting, differences in prompt engineering across studies could have contributed to the observed heterogeneity, warranting further exploration. Some biomedical disciplines could not be analyzed independently due to inconsistent reporting of outcomes (e.g., microbiology and immunology). Other disciplines had too few studies (e.g., genetics), precluding explorations of their discipline‐specific pooled accuracies. Given the literature's GPT dominance, this meta‐analysis emphasized GPT outcomes and was unable to critically evaluate other LLMs at the discipline level because of limited data. Our analysis was also limited to text‐based MCQs, as evaluating LLM accuracy on image‐based questions was beyond this study's scope.

### Future directions

The observed heterogeneity and lack of explanatory covariates, aside from LLM version, suggest that LLM performance differs across MCQ item sets and study contexts. Standardized reporting in future primary research, akin to the CONSORT‐AI guidelines for clinical trials,^55^ may better facilitate explorations of the remaining heterogeneity. Standardized reporting of LLM versions, processing dates, input selection criteria, and data quality protocols could minimize study ambiguities/heterogeneity and enhance study replicability. Specific to MCQ analyses, descriptions of input data should explicitly indicate how MCQs were developed and/or obtained. Although our results suggest that open‐access content did not affect our findings, authors should explicitly address whether their MCQs were accessible online, particularly because newer LLMs (e.g., GPT‐5, released after our study window) possess web browsing capabilities.

Research on MCQ performance in biomedical education remains predominantly focused on GPT. Comparative discipline‐specific assessments of models from Anthropic, DeepSeek, Google, and Microsoft should be broadened as data become available. The factual accuracy and pedagogical uses of custom chatbots (e.g., AnatBuddy)^56^ should be further investigated to determine whether such resources are more reliable tools to enhance student learning than commercial LLMs as they continue to advance. Such information would allow educators to make more informed decisions about when and how to integrate AI into educational activities, while considering institutional LLM availability.

Continued vigilance is warranted to monitor AI performance across disciplines and guide conversations about appropriate LLM use in medical education. While some journals now prioritize AI‐integrated pedagogy research over performance benchmarking, this shift carries risks if executed prematurely. Without vetting LLM reliability and validity in educational contexts, studies cannot distinguish whether poor outcomes reflect flawed pedagogy or unreliable AI performance. Exposing students to inaccurate AI tools risks spreading misinformation and undermining learning. Accordingly, rigorous validation studies remain indispensable for establishing the empirical foundation needed to credibly evaluate AI integration in medical education.

## CONCLUSIONS

Our meta‐analysis found a statistically significant improvement in the accuracy of OpenAI's LLMs on biomedical MCQs across successive versions. Newer GPT models (GPT‐4o through GPT o1) achieved a pooled accuracy of 90%, a substantial improvement over earlier iterations, with model progression accounting for over half of the observed performance variance. These advancements translate into a sixfold increase in the likelihood of correct responses relative to GPT‐3/3.5. The accuracy levels for Anthropic's and DeepSeek's newer models approached those of GPT‐4o_o1, while Google Gemini and Microsoft Copilot lagged behind. Newer OpenAI models (GPT‐4 through o1) achieved a pooled accuracy of ≥83% for five of the six biomedical disciplines analyzed. These improvements suggest that leveraging commercial LLMs to supplement learning in foundational biomedical sciences is becoming more practical. However, the use of LLMs should remain guided by human content experts, and educators should exercise caution, particularly when employing LLMs in more complex or image‐focused fields such as pathology. As LLM technology continues to advance, ongoing comparative evaluations will be critical for guiding best practices and maximizing the benefits of GenAI in biomedical contexts. These outcomes allow anatomy educators to apply both discipline‐specific results alongside those of other biomedical sciences, which is particularly relevant for integrated curricula.

## AUTHOR CONTRIBUTIONS

**Sharon Duffy:** Investigation; writing – review and editing. **Fathima Raahima Riyas Mohamed:** Investigation; writing – review and editing. **Robert Becker:** Investigation; writing – review and editing. **Volodymyr Mavrych:** Investigation; writing – review and editing. **Victoria Gomez:** Investigation; writing – review and editing. **Emily Rush:** Investigation; writing – review and editing. **Colleen M. Cheverko:** Methodology; data curation; investigation; formal analysis; visualization; writing – original draft; writing – review and editing. **Victoria A. Roach:** Investigation; writing – review and editing. **Jessica N. Byram:** Investigation; writing – review and editing. **Olena Bolgova:** Investigation; writing – review and editing. **Jennifer Westrick:** Investigation; writing – review and editing. **Lorena Juarez:** Investigation; writing – review and editing. **Alison F. Doubleday:** Investigation; writing – review and editing. **Joy Y. Balta:** Investigation; writing – review and editing. **Rebecca S. Lufler:** Investigation; writing – review and editing. **Jason W. Middleton:** Investigation; writing – review and editing. **Brenda K. Anak Ganeng:** Investigation; writing – review and editing. **Kathryn A. Solka:** Investigation; writing – review and editing. **Kirsten M. Brown:** Investigation; writing – review and editing. **Jason C. Mussell:** Investigation; writing – review and editing. **Michael A. Pascoe:** Investigation; writing – review and editing. **Leslie A. Hoffman:** Investigation; writing – review and editing. **Nicole DeVaul:** Investigation; writing – review and editing. **Georgina C. Stephens:** Investigation; writing – review and editing. **Colleen N. Garnett:** Investigation; writing – review and editing. **Hannah L. Herriott:** Investigation; writing – review and editing. **Adam B. Wilson:** Methodology; data curation; investigation; formal analysis; visualization; supervision; conceptualization; writing – original draft; writing – review and editing.

## FUNDING INFORMATION

No external funding was obtained for this project.

## ETHICS STATEMENT

The data presented in this manuscript were extracted from publications.

## AI DECLARATION

The authors used ChatGPT‐4.1 and Claude 4.0 Sonnet to refine and optimize phrasing for clarity and concision. The authors reviewed and edited the content and take full responsibility for the published article.

## Supporting information


Appendix S1.

Appendix S2.

Appendix S3.

Appendix S4.

## Data Availability

The data that support the findings of this study are available from the corresponding author upon reasonable request.

## References

[1] Lucas HC , Upperman JS , Robinson JR . A systematic review of large language models and their implications in medical education. Med Educ. 2024;58(11):1276–1285. 38639098 10.1111/medu.15402

[2] Turner L , Hashimoto DA , Vasisht S , Schaye V . Demystifying AI: current state and future role in medical education assessment. Acad Med. 2024;99(4S):S42. 10.1097/ACM.0000000000005598 38166201

[3] Boscardin CK , Gin B , Golde PB , Hauer KE . ChatGPT and generative artificial intelligence for medical education: potential impact and opportunity. Acad Med. 2024;99(1):22–27. 37651677 10.1097/ACM.0000000000005439

[4] Triola MM , Rodman A . Integrating generative artificial intelligence into medical education: curriculum, policy, and governance strategies. Acad Med. 2025;100(4):413–418. 39705530 10.1097/ACM.0000000000005963

[5] Yaneva V , Baldwin P , Jurich DP , Swygert K , Clauser BE . Examining ChatGPT performance on USMLE sample items and implications for assessment. Acad Med. 2024;99(2):192–197. 37934828 10.1097/ACM.0000000000005549 PMC11444356

[6] Laupichler MC , Rother JF , Grunwald Kadow IC , Ahmadi S , Raupach T . Large language models in medical education: comparing ChatGPT‐ to human‐generated exam questions. Acad Med. 2024;99(5):508–512. 38166323 10.1097/ACM.0000000000005626

[7] Ch'en PY , Day W , Pekson RC , Barrientos J , Burton WB , Ludwig AB , et al. GPT‐4 generated answer rationales to multiple choice assessment questions in undergraduate medical education. BMC Med Educ. 2025;25:333. 40038669 10.1186/s12909-025-06862-z PMC11877964

[8] Law AK , So J , Lui CT , Choi YF , Cheung KH , Kei‐Ching Hung K , et al. AI versus human‐generated multiple‐choice questions for medical education: a cohort study in a high‐stakes examination. BMC Med Educ. 2025;25(1):208. 39923067 10.1186/s12909-025-06796-6 PMC11806894

[9] Artsi Y , Sorin V , Konen E , Glicksberg BS , Nadkarni G , Klang E . Large language models for generating medical examinations: systematic review. BMC Med Educ. 2024;24:354. 38553693 10.1186/s12909-024-05239-y PMC10981304

[10] Funk PF , Hoch CC , Knoedler S , Knoedler L , Cotofana S , Sofo G , et al. ChatGPT's response consistency: a study on repeated queries of medical examination questions. Eur J Investig Health Psychol Educ. 2024;14(3):657–668. 10.3390/ejihpe14030043 PMC10969490 38534904

[11] Ra Vishal A , Harshitha AS , Venkatesh S , Abhivanth R , Pavithra MB , Suwarna M . Assessing ChatGPT's accuracy and reliability in medical education: a cross‐sectional study. Turk J Public Health. 2025;23(1):11–17.

[12] Geetha SD , Khan A , Khan A , Kannadath BS , Vitkovski T . Evaluation of ChatGPT pathology knowledge using board‐style questions. Am J Clin Pathol. 2024;161(4):393–398. 38041797 10.1093/ajcp/aqad158

[13] Koga S . Exploring the pitfalls of large language models: inconsistency and inaccuracy in answering pathology board examination‐style questions. Pathol Int. 2023;73(12):618–620. 37818818 10.1111/pin.13382

[14] Mavrych V , Ganguly P , Bolgova O . Using large language models (ChatGPT, copilot, PaLM, bard, and Gemini) in gross anatomy course: comparative analysis. Clin Anat. 2025;38(2):200–210. 39573871 10.1002/ca.24244

[15] Meo SA , Al‐Masri AA , Alotaibi M , Meo MZS , Meo MOS . ChatGPT knowledge evaluation in basic and clinical medical sciences: multiple choice question examination‐based performance. Health. 2023;11(14):2046. 10.3390/healthcare11142046 PMC10379728 37510487

[16] Al‐Khater KMK . Comparative assessment of three AI platforms in answering USMLE step 1 anatomy questions or identifying anatomical structures on radiographs. Clin Anat. 2025;38(2):186–199. 39558670 10.1002/ca.24243

[17] Agarwal M , Sharma P , Wani P . Evaluating the accuracy and reliability of large language models (ChatGPT, Claude, DeepSeek, Gemini, Grok, and Le chat) in answering item‐analyzed multiple‐choice questions on blood physiology. Cureus. 2025;17(4):e81871. 40342473 10.7759/cureus.81871 PMC12060195

[18] Bicknell BT , Butler D , Whalen S , Ricks J , Dixon CJ , Clark AB , et al. ChatGPT‐4 Omni performance in USMLE disciplines and clinical skills: comparative analysis. JMIR Med Educ. 2024;10:e63430. 39504445 10.2196/63430 PMC11611793

[19] Andrew A , Zhao S . Performance of ChatGPT versus Google bard on answering postgraduate‐level surgical examination questions: a meta‐analysis. Indian J Surg. 2025;88:22–31.

[20] Wu J , Nishida T , Liu TYA . Accuracy of large language models in answering ophthalmology board‐style questions: a meta‐analysis. Asia Pac J Ophthalmol. 2024;13(5):100106. 10.1016/j.apjo.2024.100106 39374807

[21] Wei J , Wang X , Huang M , Xu Y , Yang W . Evaluating the performance of ChatGPT on board‐style examination questions in ophthalmology: a meta‐analysis. J Med Syst. 2025;49(1):94. 40615678 10.1007/s10916-025-02227-7

[22] Jaleel A , Aziz U , Farid G , Zahid Bashir M , Mirza TR , Khizar Abbas SM , et al. Evaluating the potential and accuracy of ChatGPT‐3.5 and 4.0 in medical licensing and in‐training examinations: systematic review and meta‐analysis. JMIR Med Educ. 2025;11:e68070. 40973108 10.2196/68070 PMC12495368

[23] Jin HK , Lee HE , Kim E . Performance of ChatGPT‐3.5 and GPT‐4 in national licensing examinations for medicine, pharmacy, dentistry, and nursing: a systematic review and meta‐analysis. BMC Med Educ. 2024;24(1):1013. 39285377 10.1186/s12909-024-05944-8 PMC11406751

[24] Liu M , Okuhara T , Chang X , Shirabe R , Nishiie Y , Okada H , et al. Performance of ChatGPT across different versions in medical licensing examinations worldwide: systematic review and meta‐analysis. J Med Internet Res. 2024;26(1):e60807. 39052324 10.2196/60807 PMC11310649

[25] Levin G , Horesh N , Brezinov Y , Meyer R . Performance of ChatGPT in medical examinations: a systematic review and a meta‐analysis. BJOG. 2024;131(3):378–380. 37604703 10.1111/1471-0528.17641

[26] Moher D , Liberati A , Tetzlaff J , Altman DG . Preferred reporting items for systematic reviews and meta‐analyses: the PRISMA statement. PLoS Med. 2009;6(7):e1000097. 19621072 10.1371/journal.pmed.1000097 PMC2707599

[27] Methley AM , Campbell S , Chew‐Graham C , McNally R , Cheraghi‐Sohi S . PICO, PICOS and SPIDER: a comparison study of specificity and sensitivity in three search tools for qualitative systematic reviews. BMC Health Serv Res. 2014;14:579. 25413154 10.1186/s12913-014-0579-0 PMC4310146

[28] Landis JR , Koch GG . The measurement of observer agreement for categorical data. Biometrics. 1977;33(1):159–174. 843571

[29] Balduzzi S , Rücker G , Schwarzer G . How to perform a meta‐analysis with R: a practical tutorial. Evid Based Ment Health. 2019;22(4):153–160. 31563865 10.1136/ebmental-2019-300117 PMC10231495

[30] Borenstein M , Hedges LV , Higgins JPT &, Rothstein HR . Introduction to meta‐analysis. Hoboken, NJ: John Wiley & Sons, Ltd; 2009.

[31] Gurevitch J , Koricheva J , Nakagawa S , Stewart G . Meta‐analysis and the science of research synthesis. Nature. 2018;555(7695):175–182. 29517004 10.1038/nature25753

[32] Huedo‐Medina TB , Sánchez‐Meca J , Marín‐Martínez F , Botella J . Assessing heterogeneity in meta‐analysis: *Q* statistic or *I* ^2^ index? Psychol Methods. 2006;11(2):193–206. 16784338 10.1037/1082-989X.11.2.193

[33] Higgins JPT , Thompson SG . Quantifying heterogeneity in a meta‐analysis. Stat Med. 2002;21(11):1539–1558. 12111919 10.1002/sim.1186

[34] Higgins JPT , Thompson SG , Deeks JJ , Altman DG . Measuring inconsistency in meta‐analyses. BMJ. 2003;327(7414):557–560. 12958120 10.1136/bmj.327.7414.557 PMC192859

[35] Barker TH , Migliavaca CB , Stein C , Colpani V , Falavigna M , Aromataris E , et al. Conducting proportional meta‐analysis in different types of systematic reviews: a guide for synthesisers of evidence. BMC Med Res Methodol. 2021;21:189. 34544368 10.1186/s12874-021-01381-z PMC8451728

[36] Morton SC , Adams JL , Suttorp MJ , Shekelle PG . Meta‐regression approaches: what, why, when, and how? Rockville, MD: Agency for Healthcare Research and Quality (US); 2004.

[37] Moglia A , Georgiou K , Cerveri P , Mainardi L , Satava RM , Cuschieri A . Large language models in healthcare: from a systematic review on medical examinations to a comparative analysis on fundamentals of robotic surgery online test. Artif Intell Rev. 2024;57(9):231.

[38] Nguyen D , Kim GHJ , Bedayat A . Evaluating ChatGPT's performance across radiology subspecialties: a meta‐analysis of board‐style examination accuracy and variability. Clin Imaging. 2025;125:110551. 40561783 10.1016/j.clinimag.2025.110551

[39] Mavrych V , Yousef EM , Yaqinuddin A , Bolgova O . Large language models in medical education: a comparative cross‐platform evaluation in answering histological questions. Med Educ Online. 2025;30(1):2534065. 40651009 10.1080/10872981.2025.2534065 PMC12258195

[40] Szabó A , Laein GD . Comparative evaluation of large language models performance in medical education using urinary system histology assessment. Sci Rep. 2025;15(1):31933. 40883524 10.1038/s41598-025-17571-4 PMC12397380

[41] Bajčetić M , Mirčić A , Rakočević J , Đoković D , Milutinović K , Zaletel I . Comparing the performance of artificial intelligence learning models to medical students in solving histology and embryology multiple choice questions. Ann Anat. 2024;254:152261. 38521363 10.1016/j.aanat.2024.152261

[42] Bolgova O , Ganguly P , Mavrych V . Comparative analysis of LLMs performance in medical embryology: a cross‐platform study of ChatGPT, Claude, Gemini, and copilot. Anat Sci Educ. 2025;18(7):718–726. 40350555 10.1002/ase.70044

[43] Mavrych V , Yaqinuddin A , Bolgova O . Claude, ChatGPT, copilot, and Gemini performance versus students in different topics of neuroscience. Adv Physiol Educ. 2025;49:430–437. 39824512 10.1152/advan.00093.2024

[44] Bolgova O , Mavrych V . Evolution of AI in anatomy education study based on comparison of current large language models against historical ChatGPT performance. Sci Rep. 2025;15(1):37545. 41152541 10.1038/s41598-025-22437-w PMC12569271

[45] Saowaprut P , Wabina RS , Yang J , Siriwat L . Performance of large language models on Thailand's national medical licensing examination: a cross‐sectional study. J Educ Eval Health Prof. 2025;22:16. 40354784 10.3352/jeehp.2025.22.16

[46] Monroe CL , Abdelhafez YG , Atsina K , Aman E , Nardo L , Madani MH . Evaluation of responses to cardiac imaging questions by the artificial intelligence large language model ChatGPT. Clin Imaging. 2024;112:110193. 38820977 10.1016/j.clinimag.2024.110193

[47] Zhu L , Mou W , Lai Y , Chen J , Lin S , Xu L , et al. Step into the era of large multimodal models: a pilot study on ChatGPT‐4 V(ision)'s ability to interpret radiological images. Int J Surg. 2024;110(7):4096–4102. 38498394 10.1097/JS9.0000000000001359 PMC11254196

[48] Hyeamang LJ , Sekhar TC , Rush E , Beresheim AC , Cheverko CM , Brooks WS , et al. AI's ability to interpret unlabeled anatomy images and supplement educational research as an AI rater. Anat Sci Educ. 2025;18(10):1102–1113. 40646660 10.1002/ase.70074 PMC12511656

[49] Masters K , MacNeil H , Benjamin J , Carver T , Nemethy K , Valanci‐Aroesty S , et al. Artificial intelligence in health professions education assessment: AMEE guide no. 178. Med Teach. 2025;47(9):1410–1424. 39787028 10.1080/0142159X.2024.2445037

[50] Xu T , Weng H , Liu F , Yang L , Luo Y , Ding Z , et al. Current status of ChatGPT use in medical education: potentials, challenges, and strategies. J Med Internet Res. 2024;26(1):e57896. 39196640 10.2196/57896 PMC11391159

[51] Cheng Y , Zhu L . A review of ChatGPT in medical education: exploring advantages and limitations. Int J Surg. 2025;111(7):4586–4602. 40465793 10.1097/JS9.0000000000002505

[52] Zhui L , Yhap N , Liping L , Zhengjie W , Zhonghao X , Xiaoshu Y , et al. Impact of large language models on medical education and teaching adaptations. JMIR Med Inform. 2024;12:e55933. 39087590 10.2196/55933 PMC11294775

[53] Tangsrivimol JA , Darzidehkalani E , Virk HUH , Wang Z , Egger J , Wang M , et al. Benefits, limits, and risks of ChatGPT in medicine. Front Artif Intell. 2025;8:1518049. 39949509 10.3389/frai.2025.1518049 PMC11821943

[54] Rush E , Byram JN , Garnett CN , DeVaul N , Smith L , Checchi M , et al. An audit of AI‐related documents across U.S. medical schools: a framework‐based qualitative content analysis. Med Teach. 2025;48(3):493–505. 41021415 10.1080/0142159X.2025.2564869

[55] Liu X , Cruz Rivera S , Moher D , Calvert MJ , Denniston AK . Reporting guidelines for clinical trial reports for interventions involving artificial intelligence: the CONSORT—AI extension. Nat Med. 2020;26(9):1364–1374. 32908283 10.1038/s41591-020-1034-x PMC7598943

[56] Arun G , Perumal V , Urias FPJB , Ler YE , Tan BWT , Vallabhajosyula R , et al. ChatGPT versus a customized AI chatbot (Anatbuddy) for anatomy education: a comparative pilot study. Anat Sci Educ. 2024;17(7):1396–1405. 39169464 10.1002/ase.2502

