# Supplementary material for: The performance of ChatGPT and other large language models on multiple‐choice questions in biomedical disciplines: A meta‐analysis
Source: Anat Sci Educ. 2026 May 19;19(9):1556–69. doi: 10.1002/ase.70262 (PMC13545332; doi:10.1002/ase.70262)
Supplement: Supplementary file 1 [file ASE-19-1556-s002.docx]

**Search Strategy**

By Jennifer C Westrick, MSc, MSLIS, AHIP

Search strategy overview

Articles had to have a term from the first set below, as well as a term from the second set that was within five words of a term from the third set. All terms had to be in the title or abstract.

(ChatGPT OR "Generative pre-trained transformer*" OR "generative model*" OR "genAI" OR "Large Language Model*" OR LLM OR GPT OR Claude OR Gemini OR PaLM OR Copilot OR Llama OR perplexity OR DeepSeek OR Bard OR Qwen OR Grok OR Mistral OR PaLM OR Inflection OR Phi OR Gemma OR "Bing Chat" OR "Large Language Models"[Mesh])

AND (("Audiology” OR "Audiologist” OR "Anesthesiology” OR "Anesthesiologist” OR "Anesthetists” OR "Dental” OR "Dentist” OR "Dentistry” OR "Endodontist” OR "Emergency” OR "Imaging” OR "Maxillofacial” OR "Nurse” OR "Nurses” OR "Nursing” OR "Nutrition” OR "Nutritionist” OR "Occupational” OR "Ophthalmic” OR "Ophthalmology” OR "Ophthalmologist” OR "Optician” OR "Optometry” OR "Optometrist” OR "Orthodontist” OR "Orthotics” OR "Oral Surgeon” OR "Paramedicine” OR "Paramedics” OR "Pediatric Assistant” OR "Pharmacy” OR "Pharmacist” OR "Physical Therapy” OR "Physical Therapist” OR "Physician Assistant” OR "Podiatry” OR "Podiatrist” OR "Prosthetics” OR "Respiratory Therapy” OR "Respiratory Therapist” OR "Speech Language Pathology” OR "Speech Language Pathologist” OR "medical”)

Within five words of (certification OR license OR board OR “multiple choice")

Reproducible PubMed search:

(ChatGPT[Title/Abstract] OR "Generative pre-trained transformer*"[Title/Abstract] OR "generative model*"[Title/Abstract] OR "genAI"[Title/Abstract] OR "Large Language Model*"[Title/Abstract] OR LLM[Title/Abstract] OR GPT[Title/Abstract] OR Claude[Title/Abstract] OR Gemini[Title/Abstract] OR PaLM[Title/Abstract] OR Copilot[Title/Abstract] OR Llama[Title/Abstract] OR perplexity[Title/Abstract] OR DeepSeek[Title/Abstract] OR Bard[Title/Abstract] OR Qwen[Title/Abstract] OR Grok[Title/Abstract] OR Mistral[Title/Abstract] OR PaLM[Title/Abstract] OR Inflection[Title/Abstract] OR Phi[Title/Abstract] OR Gemma[Title/Abstract] OR "Bing Chat"[Title/Abstract] OR "Large Language Models"[Mesh])

AND (("Multiple-choice*"[Title/Abstract] OR MCQ[Title/Abstract] OR MCQs[Title/Abstract] OR USMLE*[Title/Abstract] OR "medical licens*"[Title/Abstract] OR "question format*"[Title/Abstract] OR "multiple response*"[Title/Abstract] OR "exam question*"[Title/Abstract] OR "examination question*"[Title/Abstract] OR "assessment question*"[Title/Abstract] OR "certification exam*"[Title/Abstract] OR “medical school exam*”[Title/Abstract] OR “licensing exam*”[Title/Abstract])

OR ("Audiology certification" [tiab:~5] OR "Audiologist certification" [tiab:~5] OR "Anesthesiology certification" [tiab:~5] OR "Anesthesiologist certification" [tiab:~5] OR "Anesthetists certification" [tiab:~5] OR "Dental certification" [tiab:~5] OR "Dentist certification" [tiab:~5] OR "Dentistry certification" [tiab:~5] OR "Endodontist certification" [tiab:~5] OR "Emergency certification" [tiab:~5] OR "Imaging certification" [tiab:~5] OR "Maxillofacial certification" [tiab:~5] OR "Nurse certification" [tiab:~5] OR "Nurses certification" [tiab:~5] OR "Nursing certification" [tiab:~5] OR "Nutrition certification" [tiab:~5] OR "Nutritionist certification" [tiab:~5] OR "Occupational certification" [tiab:~5] OR "Ophthalmic certification" [tiab:~5] OR "Ophthalmology certification" [tiab:~5] OR "Ophthalmologist certification" [tiab:~5] OR "Optician certification" [tiab:~5] OR "Optometry certification" [tiab:~5] OR "Optometrist certification" [tiab:~5] OR "Orthodontist certification" [tiab:~5] OR "Orthotics certification" [tiab:~5] OR "Oral Surgeon certification" [tiab:~5] OR "Paramedicine certification" [tiab:~5] OR "Paramedics certification" [tiab:~5] OR "Pediatric Assistant certification" [tiab:~5] OR "Pharmacy certification" [tiab:~5] OR "Pharmacist certification" [tiab:~5] OR "Physical Therapy certification" [tiab:~5] OR "Physical Therapist certification" [tiab:~5] OR "Physician Assistant certification" [tiab:~5] OR "Podiatry certification" [tiab:~5] OR "Podiatrist certification" [tiab:~5] OR "Prosthetics certification" [tiab:~5] OR "Respiratory Therapy certification" [tiab:~5] OR "Respiratory Therapist certification" [tiab:~5] OR "Speech Language Pathology certification" [tiab:~5] OR "Speech Language Pathologist certification" [tiab:~5] OR "medical certification" [tiab:~5]

OR "Audiology license" [tiab:~5] OR "Audiologist license" [tiab:~5] OR "Anesthesiology license" [tiab:~5] OR "Anesthesiologist license" [tiab:~5] OR "Anesthetists license" [tiab:~5] OR "Dental license" [tiab:~5] OR "Dentist license" [tiab:~5] OR "Dentistry license" [tiab:~5] OR "Endodontist license" [tiab:~5] OR "Emergency license" [tiab:~5] OR "Imaging license" [tiab:~5] OR "Maxillofacial license" [tiab:~5] OR "Nurse license" [tiab:~5] OR "Nurses license" [tiab:~5] OR "Nursing license" [tiab:~5] OR "Nutrition license" [tiab:~5] OR "Nutritionist license" [tiab:~5] OR "Occupational license" [tiab:~5] OR "Ophthalmic license" [tiab:~5] OR "Ophthalmology license" [tiab:~5] OR "Ophthalmologist license" [tiab:~5] OR "Optician license" [tiab:~5] OR "Optometry license" [tiab:~5] OR "Optometrist license" [tiab:~5] OR "Orthodontist license" [tiab:~5] OR "Orthotics license" [tiab:~5] OR "Oral Surgeon license" [tiab:~5] OR "Paramedicine license" [tiab:~5] OR "Paramedics license" [tiab:~5] OR "Pediatric Assistant license" [tiab:~5] OR "Pharmacy license" [tiab:~5] OR "Pharmacist license" [tiab:~5] OR "Physical Therapy license" [tiab:~5] OR "Physical Therapist license" [tiab:~5] OR "Physician Assistant license" [tiab:~5] OR "Podiatry license" [tiab:~5] OR "Podiatrist license" [tiab:~5] OR "Prosthetics license" [tiab:~5] OR "Respiratory Therapy license" [tiab:~5] OR "Respiratory Therapist license" [tiab:~5] OR "Speech Language Pathology license" [tiab:~5] OR "Speech Language Pathologist license" [tiab:~5] OR "medical license" [tiab:~5]

OR "Audiology board" [tiab:~5] OR "Audiologist board" [tiab:~5] OR "Anesthesiology board" [tiab:~5] OR "Anesthesiologist board" [tiab:~5] OR "Anesthetists board" [tiab:~5] OR "Dental board" [tiab:~5] OR "Dentist board" [tiab:~5] OR "Dentistry board" [tiab:~5] OR "Endodontist board" [tiab:~5] OR "Emergency board" [tiab:~5] OR "Imaging board" [tiab:~5] OR "Maxillofacial board" [tiab:~5] OR "Nurse board" [tiab:~5] OR "Nurses board" [tiab:~5] OR "Nursing board" [tiab:~5] OR "Nutrition board" [tiab:~5] OR "Nutritionist board" [tiab:~5] OR "Occupational board" [tiab:~5] OR "Ophthalmic board" [tiab:~5] OR "Ophthalmology board" [tiab:~5] OR "Ophthalmologist board" [tiab:~5] OR "Optician board" [tiab:~5] OR "Optometry board" [tiab:~5] OR "Optometrist board" [tiab:~5] OR "Orthodontist board" [tiab:~5] OR "Orthotics board" [tiab:~5] OR "Oral Surgeon board" [tiab:~5] OR "Paramedicine board" [tiab:~5] OR "Paramedics board" [tiab:~5] OR "Pediatric Assistant board" [tiab:~5] OR "Pharmacy board" [tiab:~5] OR "Pharmacist board" [tiab:~5] OR "Physical Therapy board" [tiab:~5] OR "Physical Therapist board" [tiab:~5] OR "Physician Assistant board" [tiab:~5] OR "Podiatry board" [tiab:~5] OR "Podiatrist board" [tiab:~5] OR "Prosthetics board" [tiab:~5] OR "Respiratory Therapy board" [tiab:~5] OR "Respiratory Therapist board" [tiab:~5] OR "Speech Language Pathology board" [tiab:~5] OR "Speech Language Pathologist board" [tiab:~5] OR "medical board exam" [tiab:~5] OR "medical board examination" [tiab:~5]

OR "Audiology multiple choice" [tiab:~5] OR "Audiologist multiple choice" [tiab:~5] OR "Anesthesiology multiple choice" [tiab:~5] OR "Anesthesiologist multiple choice" [tiab:~5] OR "Anesthetists multiple choice" [tiab:~5] OR "Dental multiple choice" [tiab:~5] OR "Dentist multiple choice" [tiab:~5] OR "Dentistry multiple choice" [tiab:~5] OR "Endodontist multiple choice" [tiab:~5] OR "Emergency multiple choice" [tiab:~5] OR "Imaging multiple choice" [tiab:~5] OR "Maxillofacial multiple choice" [tiab:~5] OR "Nurse multiple choice" [tiab:~5] OR "Nurses multiple choice" [tiab:~5] OR "Nursing multiple choice" [tiab:~5] OR "Nutrition multiple choice" [tiab:~5] OR "Nutritionist multiple choice" [tiab:~5] OR "Occupational multiple choice" [tiab:~5] OR "Ophthalmic multiple choice" [tiab:~5] OR "Ophthalmology multiple choice" [tiab:~5] OR "Ophthalmologist multiple choice" [tiab:~5] OR "Optician multiple choice" [tiab:~5] OR "Optometry multiple choice" [tiab:~5] OR "Optometrist multiple choice" [tiab:~5] OR "Orthodontist multiple choice" [tiab:~5] OR "Orthotics multiple choice" [tiab:~5] OR "Oral Surgeon multiple choice" [tiab:~5] OR "Paramedicine multiple choice" [tiab:~5] OR "Paramedics multiple choice" [tiab:~5] OR "Pediatric Assistant multiple choice" [tiab:~5] OR "Pharmacy multiple choice" [tiab:~5] OR "Pharmacist multiple choice" [tiab:~5] OR "Physical Therapy multiple choice" [tiab:~5] OR "Physical Therapist multiple choice" [tiab:~5] OR "Physician Assistant multiple choice" [tiab:~5] OR "Podiatry multiple choice" [tiab:~5] OR "Podiatrist multiple choice" [tiab:~5] OR "Prosthetics multiple choice" [tiab:~5] OR "Respiratory Therapy multiple choice" [tiab:~5] OR "Respiratory Therapist multiple choice" [tiab:~5] OR "Speech Language Pathology multiple choice" [tiab:~5] OR "Speech Language Pathologist multiple choice" [tiab:~5]) )

AND (2022:3000/12/12[pdat])
