# Supplementary material for: The performance of ChatGPT and other large language models on multiple‐choice questions in biomedical disciplines: A meta‐analysis
Source: Anat Sci Educ. 2026 May 19;19(9):1556–69. doi: 10.1002/ase.70262 (PMC13545332; doi:10.1002/ase.70262)
Supplement: Supplementary file 2 [file ASE-19-1556-s003.docx]

**Figure S3.1**. OpenAI GPT-3 and GPT-3.5 results. All disciplines are included in this plot, with the disciplines listed for reference.

**Figure S3.2**. OpenAI GPT-4 results. All disciplines are included in this plot, with the disciplines listed for reference.

**Figure S3.3**. OpenAI GPT-4o through GPT o1 results. All disciplines are included in this plot, with the disciplines listed for reference.

**Figure S3.4**. OpenAI results for biochemistry.

**Figure S3.5**. OpenAI results for microbiology and immunology. The subdiscipline is listed for reference.

**Figure S3.6**. OpenAI results for pathology.

**Figure S3.7**. OpenAI results for pharmacology.

**Figure S3.8**. OpenAI results for physiology.

**Figure S3.9**. OpenAI results for USMLE Step 1 and Equivalent question sets. The studies represented in this figure did not subdivide their data by specific disciplines.

**Figure S3.10**. Results from newer LLM versions of Anthropic (Claude 3 through 3.7), DeepSeek (R1), Google (Gemini series), and Microsoft (Copilot). These newer LLM versions were included based on comparability with newer OpenAI ChatGPT versions. Discipline-specific analyses were not possible for these LLM companies because of small sample sizes.
