# Supplementary material for: The performance of ChatGPT and other large language models on multiple‐choice questions in biomedical disciplines: A meta‐analysis
Source: Anat Sci Educ. 2026 May 19;19(9):1556–69. doi: 10.1002/ase.70262 (PMC13545332; doi:10.1002/ase.70262)
Supplement: Supplementary file 3 [file ASE-19-1556-s004.docx]

**Table S4.1.** Data values supporting Figure 2.

|  | **GPT-3/3.5** | **GPT-4** | **GPT-4o to GPT o1** |
| --- | --- | --- | --- |
| **Across All Specialties*** |  |  |  |
| k: number of studies | 22 | 22 | 8 |
| q: number of question sets investigated | 67 | 49 | 21 |
| n: number of MCQs investigated | 7,627 | 4,382 | 996 |
| Pooled effect (proportion) | 0.593 | 0.819 | 0.901 |
| 95% confidence interval | 0.542-0.643 | 0.784-0.850 | 0.880-0.919 |
| p-value; Q-statistic | <0.001; 908.28 | <0.001; 264.22 | 0.954; 10.70 |
| I^2^ | 92.7% | 81.8% | 0.0% |
|  | **GPT-3/3.5** | **GPT-4 to GPT o1** |  |
| **Anatomical Sciences, including Gross Anatomy, Histology, Cell Biology, Embryology, and Neuroscience** | | | |
| k: number of studies | 9 | 9 |  |
| q: number of question sets investigated | 14 | 16 |  |
| n: number of MCQs investigated | 943 | 867 |  |
| Pooled effect (proportion) | 0.632 | 0.866 |  |
| 95% confidence interval | 0.571-0.689 | 0.813-0.906 |  |
| p-value; Q-statistic | 0.002; 32.03 | 0.002; 36.11 |  |
| I^2^ | 59.4% | 58.5% |  |
| **Biochemistry** |  |  |  |
| k: number of studies | 6 | 5 |  |
| q: number of question sets investigated | 7 | 7 |  |
| n: number of MCQs investigated | 142 | 325 |  |
| Pooled effect (proportion) | 0.629 | 0.851 |  |
| 95% confidence interval | 0.485-0.753 | 0.807-0.886 |  |
| p-value; Q-statistic | 0.028; 14.11 | 0.0948; 1.66 |  |
| I^2^ | 57.5% | 0.0% |  |
| **Microbiology and Immunology** |  |  |  |
| k: number of studies | 3 | 4 |  |
| q: number of question sets investigated | 4 | 8 |  |
| n: number of MCQs investigated | 104 | 223 |  |
| Pooled effect (proportion) | 0.500 | 0.840 |  |
| 95% confidence interval | 0.362-0.637 | 0.783-0.884 |  |
| p-value; Q-statistic | 0.128; 5.68 | 0.517; 6.19 |  |
| I^2^ | 47.2% | 0.0% |  |
| **Pathology** |  |  |  |
| k: number of studies | 5 | 6 |  |
| q: number of question sets investigated | 6 | 7 |  |
| n: number of MCQs investigated | 286 | 651 |  |
| Pooled effect (proportion) | 0.648 | 0.746 |  |
| 95% confidence interval | 0.501-0.765 | 0.630-0.835 |  |
| p-value; Q-statistic | 0.018; 13.66 | <0.001; 40.56 |  |
| I^2^ | 63.4% | 85.2% |  |
| **Pharmacology** |  |  |  |
| k: number of studies | 4 | 4 |  |
| q: number of question sets investigated | 5 | 7 |  |
| n: number of MCQs investigated | 101 | 227 |  |
| Pooled effect (proportion) | 0.613 | 0.865 |  |
| 95% confidence interval | 0.514-0.703 | 0.811-0.905 |  |
| p-value; Q-statistic | 0.864; 1.28 | 0.460; 5.68 |  |
| I^2^ | 0.0% | 0.0% |  |
| **Physiology** |  |  |  |
| k: number of studies | 6 | 4 |  |
| q: number of question sets investigated | 7 | 5 |  |
| n: number of MCQs investigated | 340 | 123 |  |
| Pooled effect (proportion) | 0.645 | 0.830 |  |
| 95% confidence interval | 0.501-0.767 | 0.626-0.935 |  |
| p-value; Q-statistic | <0.001; 24.47 | 0.003; 16.46 |  |
| I^2^ | 75.5% | 75.7% |  |
| **USMLE Step 1 and Equivalent** |  |  |  |
| k: number of studies | 8 | 8 |  |
| q: number of question sets investigated | 16 | 9 |  |
| n: number of MCQs investigated | 5,233 | 2,360 |  |
| Pooled effect (proportion) | 0.517 | 0.878 |  |
| 95% confidence interval | 0.402-0.630 | 0.831-0.914 |  |
| p-value; Q-statistic | <0.001; 676.32 | <0.001; 52.93 |  |
| I^2^ | 97.8% | 84.9% |  |

*Behavioral and Social Sciences, Bioethics, Biostatistics/statistics, and Genetics were excluded as specialties from this table because they were represented by < 5 studies. These data were included in the global analysis across all specialties.

**Table S4.2.** Data values supporting Figure 3.

| **Anthropic Claude 3 through Claude 3.7** |  |
| --- | --- |
| k: number of studies | 4 |
| q: number of question sets investigated | 13 |
| n: number of MCQs investigated | 538 |
| Pooled effect (proportion) | 0.862 |
| 95% confidence interval | 0.817-0.897 |
| p-value; Q-statistic | 0.304; 13.95 |
| I^2^ | 14.0% |
| **DeepSeek R1** |  |
| k: number of studies | 3 |
| q: number of question sets investigated | 7 |
| n: number of MCQs investigated | 345 |
| Pooled effect (proportion) | 0.860 |
| 95% confidence interval | 0.780-0.903 |
| p-value; Q-statistic | 0.278; 7.49 |
| I^2^ | 19.9% |
| **Google Gemini Series** |  |
| k: number of studies | 10 |
| q: number of question sets investigated | 23 |
| n: number of MCQs investigated | 1,093 |
| Pooled effect (proportion) | 0.755 |
| 95% confidence interval | 0.680-0.817 |
| p-value; Q-statistic | <0.001; 89.74 |
| I^2^ | 75.5% |
| **Microsoft CoPilot** |  |
| k: number of studies | 5 |
| q: number of question sets investigated | 5 |
| n: number of MCQs investigated | 850 |
| Pooled effect (proportion) | 0.694 |
| 95% confidence interval | 0.540-0.814 |
| p-value; Q-statistic | <0.001; 66.93 |
| I^2^ | 94.0% |
